# The Roles of Dehumanization and Moral Outrage in Retributive Justice

**DOI:** 10.1371/journal.pone.0061842

**Published:** 2013-04-23

**Authors:** Brock Bastian, Thomas F. Denson, Nick Haslam

**Affiliations:** 1 School of Psychology, University of Queensland, Brisbane, Queensland, Australia; 2 School of Psychology, University of New South Wales, Sydney, Australia; 3 School of Psychological Sciences, University of Melbourne, Melbourne, Australia; George Mason University/Krasnow Institute for Advanced Study, United States of America

## Abstract

When innocents are intentionally harmed, people are motivated to see that offenders get their “just deserts”. The severity of the punishment they seek is driven by the perceived magnitude of the harm and moral outrage. The present research extended this model of retributive justice by incorporating the role of offender dehumanization. In three experiments relying on survey methodology in Australia and the United States, participants read about different crimes that varied by type (child molestation, violent, or white collar – Studies 1 and 2) or severity (Study 3). The findings demonstrated that both moral outrage and dehumanization predicted punishment independently of the effects of crime type or crime severity. Both moral outrage and dehumanization mediated the relationship between perceived harm and severity of punishment. These findings highlight the role of offender dehumanization in punishment decisions and extend our understanding of processes implicated in retributive justice.

## Introduction

When criminal behavior brings harm to innocent people it has the capacity to arouse strong affective responses in third-party observers. Consider Bill Clare, who was found guilty of repeatedly raping a 6 year old girl and her 3 year old brother. The 3 year old died from the associated trauma [1]. He was sentenced to 39 years in prison and was the target of renewed calls for the death penalty for pedophiles [2]. Just the thought of Clare’s crime evokes a visceral response, not only to the criminal act, but to Clare himself.

Efforts to understand psychological responses to criminal behavior have generated a large body of knowledge within the fields of social cognition and law [3]–[10]. For example, when criminal behavior is seen as intentional (e.g., [11]–[13]), perpetrators are judged as more culpable, responsible, and blameworthy [14]–[19] and are punished more severely [20]. In these cases, when mitigating factors are scarce and crimes are viewed as intentional, people tend to endorse retributive forms of punishment [21], [22] and are highly sensitive to the harm done in forming judgements about punishment severity [22], [23].

This “just deserts” approach to punishment is grounded on the belief that offenders should be punished proportionately to the moral offensiveness and harmfulness of their crimes. Any future consequences of punishment (i.e., such as rehabilitation) become irrelevant [21]. An important factor in translating perceptions of harmfulness into recommendations for harsh punishment is the moral outrage that people feel in response to criminal acts [20]. People respond to moral transgressions with gut-level emotional responses [24]–[26] and these emotional responses play a central role in how people react to, and reason about, morally relevant behavior. Cross-cultural evidence highlights that feelings of contempt, anger, and disgust are specifically associated with these “third-party” responses to moral transgressions [27]. Providing empirical support for the role of moral outrage in punishment, Carlsmith et al. [23] found that moral outrage mediated the effects of perceived harm on the severity of recommended punishment.

Feelings of moral outrage play an important role in determining punishment severity, but other factors may also play a role. In the current research we explored one such factor – dehumanization of the offender. Anecdotal evidence supports a link between dehumanization and punishment severity. For example, the use of dehumanizing language in victim impact statements is associated with the harshness of sentencing decisions by jurors [9]. Likewise, analyses of news articles about Black American offenders suggest an association between a portrayal of Black criminals as ape-like and likelihood to be executed by the state [28]. Theoretically, viewing others as lacking core human capacities and likening them to animals or objects [29] may make them seem less sensitive to pain, more dangerous and uncontrollable, and thus more needful of severe and coercive forms of punishment [30]–[32].

A series of studies investigating reactions to sex offenders provided some initial support for these possibilities [33]. This research found that likening sex-offenders to animals was positively correlated with endorsement of harsher punishment, reduced support for rehabilitation, exclusion from society, and support for violent treatment (e.g., castration). Although this previous work links dehumanization to support for harsh punishment, it has not been incorporated into models of retributive justice (i.e., [23]) or extended to other types of crime beyond sex offenses. Furthermore, Viki et al.’s [33] pioneering work did not examine how dehumanization may be related to moral outrage, and whether, like moral outrage, it may mediate the relationship between perceived harm and punishment severity.

We argue that both moral outrage and dehumanization of offenders may arise in response to morally reprehensible behavior and that both may independently influence punishment severity. Moreover, these two responses to criminal behavior may also be related to one another. Previous work suggests that moral emotions may be linked to dehumanization in a variety of ways. Viewing others as less human reduces feelings of guilt in harm doers and reduces reparations for past wrongdoings [34]. Dehumanizing others also facilitates moral disengagement from one’s actions [35]–[41]. This prior research indicates a link between dehumanization and self-focused emotional responses to our own immoral actions (e.g., guilt and shame). In the current studies, we examined the untested notion that experiencing moral outrage (e.g., disgust, anger and contempt) in response to others’ immoral actions would be associated with reduced perceptions of their humanity. In this way, just as past work has examined dehumanization as motivated by a desire to morally disengage from one’s own actions or those of one’s group, we examine dehumanization as motivated by a perception of others harmful and morally reprehensible behavior.

Indirect evidence for a positive link between moral outrage and dehumanization comes from research showing that feelings of disgust are associated with the dehumanization of others. For instance, people high in disgust sensitivity dehumanize immigrants to a greater extent than those low in disgust sensitivity [42]. Other work has demonstrated that viewing members of marginalized outgroups known to elicit disgust (e.g., the homeless, drug addicts) does not influence activation in the medial prefrontal cortex. The medial prefrontal cortex is broadly implicated in social cognition. For instance, this region is typically activated when viewing people but not objects. Lack of activation is consistent with viewing these people as less than human. Moreover, viewing these marginalized outgroup members increased activation in the insula and amygdala, a neural pattern consistent with experiencing disgust [43]. Together, this work indicates that the emotion of disgust may be associated with perceiving others as less human (see also [44]–[46]); however, whether emotional reactions to criminal behavior are associated with dehumanized perceptions of perpetrators and whether this dehumanization is related to punishment severity remain empirical questions.

### The Current Research

The current studies investigated the association between moral outrage, dehumanization, and retributive justice. We predicted that moral outrage and dehumanization would covary in response to criminal behavior and this would be evident across a range of crimes and independent of crime severity (Hypothesis 1). Consistent with the findings of Carlsmith et al. [23], we predicted that moral outrage would be associated with punishment severity (i.e., harsher sentencing and less support for rehabilitation: Hypothesis 2). Extending this work, and consistent with our focus on dehumanization, we also predicted that offender dehumanization would predict punishment severity, and that this would occur independently of any effects associated with moral outrage (Hypothesis 3). We also predicted that, consistent with Carlsmith et al. [23], moral outrage would mediate the effects of the perceived harmfulness of the offense on severity of punishment (Hypothesis 4). Finally, extending on that work, we predicted that dehumanization would also mediate this relationship, and would do so independently of the effects of moral outrage (Hypothesis 5).

### Study 1

Study 1 tested the prediction that moral outrage and dehumanization would covary in response to criminal behavior and that the relationship between the two constructs would occur independent of crime type (Hypothesis 1). We constructed vignettes for three different types of crime: white collar, violent, and child molestation. We then measured Australian students’ emotional reactions and their perceptions of the humanity of each type of offender.

## Methods

### Participants and Design

All studies were approved by the Human Research Ethics Committee of the University of New South Wales. All participants gave informed written consent. A total of 100 first year psychology students from The University of Queensland and the University of New South Wales participated in the study in exchange for course credit (58 women, 60% Asian, 25% Australian, 8% European and 6% other nationality, *M_age_* = 22.74, *SD* = 5.21). In the laboratory, participants were randomly assigned to read about either a violent crime (*n* = 35), white collar crime (*n* = 33), or a child molestation crime (*n* = 32). Each crime condition contained two different vignettes which were alternated between participants to ensure a broad sampling of crime types. Participants were told the study involved perceptions of criminals.

### Materials and Procedure

#### Crime descriptions

After completing demographic questions, participants read one of the crime vignettes (see Appendix S1). The descriptions were adapted from news stories found online; names and identifying details were changed. Each vignette had approximately equal amounts of detail, including information about the criminal’s age, origin, the crime, and the consequences. For example, one violent crime vignette described a 48-year old man who “hacked to death 7 young children and 2 adults with a meat cleaver in rural Victoria.” The child molester vignettes include a description of a 53-year old pediatrician from Perth who “used his practice to molest boys and teenagers”. One of the white collar crime vignettes describes a 34-year old Brisbane man who “fleeced almost $127,000 from family and friends by stealing money they had given to him for investments”.

#### Moral outrage

Participants completed questionnaires after reading their designated crime description. The first questionnaire consisted of 10 emotion descriptors which comprised 3 different subscales measuring disgust, anger, and contempt. These classifications were based on Haidt’s [47] work on moral outrage emotions and prior research [48]. Participants were instructed to indicate on a scale from 1 (not at all) to 7 (extremely so) the degree to which they felt each emotion when considering the crime they just read about. The subscale measuring Disgust (α = .92, *M = *4.11, *SD* = 1.81) included the items “grossed out”, “disgusted”, “queasy”, and “sick to my stomach”. The subscale measuring Anger (α = .94, *M = *4.33, *SD* = 1.88) included the items “angry”, “mad” and “furious”. The subscale for Contempt (α = .92, *M = *3.90, *SD* = 1.67) included “contempt”, “disdain” and “scorn”.

#### Dehumanization

Participants were asked to consider the criminal in the description and rated agreement with 8 statements (1 = not at all, 7 = extremely so) taken from Bastian and Haslam [49] assessing the denial of Human Nature (4-items; e.g., “I felt like the person in the story was open minded, like they could think clearly about things” [*reversed*], “I felt like the person in the story was emotional, like they were responsive and warm” [*reversed*], “I felt like the person in the story was superficial like they had no depth”, “I felt like the person in the story was mechanical and cold, like a robot”) and denial of Human Uniqueness (4-items; e.g., “I felt like the person in the story was refined and cultured” [*reversed*], “I felt like the person in the story was rational and logical, like they were intelligent” [*reversed*], “I felt like the person in the story lacked self-restraint, like an animal”, “I felt like the person in the story was unsophisticated”).

## Results and Discussion

### Moral Outrage

**Figure 1:**
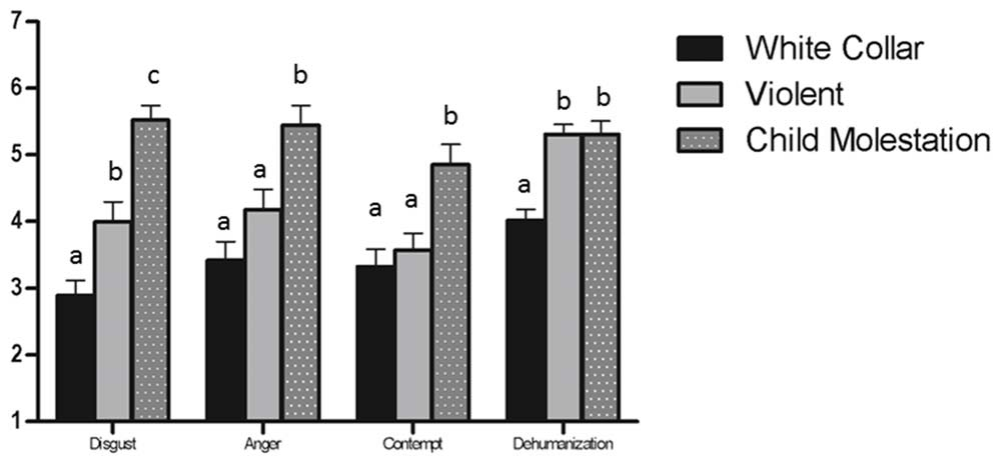
Mean differences in moral emotions and perceived humanness across crime types, Study 1. Note: Values with different superscripts are significantly (*p*<.05) different from each other controlling for familywise error (Scheffé’s test).

A one-way ANOVA revealed a significant effect of crime type on each of the moral outrage emotions: Disgust, *F*(2,96) = 26.35, *p*<.01, η^2^ = .35; Anger, *F*(2,97) = 11.64, *p*<.01, η^2^ = .19; and Contempt, *F*(2,96) = 9.05, *p*<.01, η^2^ = .16. Post hoc comparisons (see Figure 1) revealed that child molestation crimes produced more anger, contempt, and disgust that either violent crimes or white collar crimes. Violent crimes also produced more disgust that white collar crimes.

### Dehumanization

Principal components analysis indicated that the measure of dehumanization formed a single factor solution explaining 38.45% of the variance, with all items loading above.45. The two dimensions of humanness have been examined separately in other work (see [29] for a review). However, when examining person perception within the context of harmful behavior, these two dimensions consistently either form a single dimension (e.g., [50], [51]) or both dimensions show similar patterns of effects (e.g., [49]). This lack of differentiation, suggests that perceptions of dehumanization may operate somewhat differently when they arise in response to the harmful behavior of individuals rather than in judgments of groups (see [52]). Thus, a single dehumanization scale was constructed (α = .77, *M = *3.12, *SD = *1.15). One-way ANOVAs revealed a main effect of the type of crime on dehumanization, *F*(2,97) = 19.34, *p*<.01, η^2^ = .29 Post hoc comparisons (see Figure 1) revealed that dehumanization was lower for white collar crimes compared to violent and child molestation crimes.

### Correlates of Dehumanization

Table 1 shows the correlates of dehumanization for the entire sample. All of the moral outrage emotions were positively associated with dehumanization. Hierarchical regression analyses predicting dehumanization revealed no crime × emotion interactions, suggesting that the relationship between moral outrage emotions and dehumanization was uniform across the types of crimes, *t*s<1.

**Table 1:** Zero-order correlations between dehumanization and moral emotions in Studies 1, 2, and 3.

	Dehumanization	Disgust	Anger
Study 1			
Disgust	.37***		
Anger	.35***	.80***	
Contempt	.29**	.49***	.59***
Study 2			
Disgust	.31**		
Anger	.29**	.76***	
Contempt	.21*	.62***	.65***
Study 3			
Disgust	.30***		
Anger	.35***	.64***	
Contempt	.37***	.57***	.60***

NOTE: ***p<.001,

** *p*<.01,

* *p*<.05.

Study 1 provided initial evidence that although mean levels of moral outrage and dehumanization varied across crime types, the relationship between moral outrage and dehumanization of offenders was equivalent across crime types (Hypothesis 1). Specifically, increased moral outrage was uniformly associated with increased dehumanization of the offenders regardless of crime type.

### Study 2

In Study 2 we expected to replicate our findings showing the positive relationships between moral outrage and offender dehumanization observed in Study 1 (Hypothesis 1). Study 2 also extended Study 1 by including measures of sentencing and support for rehabilitation, therefore allowing us to investigate links between moral outrage, dehumanization and retributive justice. We predicted that both moral outrage (Hypothesis 2) and dehumanization (Hypothesis 3) would be related to punishment severity. Thus, we expected that increased moral outrage and greater offender dehumanization would be associated with harsher sentencing, and less support for rehabilitation. In addition, we predicted that these effects would occur independently of any effects associated with crime type (Hypothesis 1). Finally, we also included a measure of offender blame. One might hypothesize that dehumanized criminals may be *less blameworthy* than criminals viewed as more human. Dehumanized criminals might be perceived as less able to control themselves and therefore less responsible [16]. We argue, however, that in responses to criminal behavior, blame and dehumanization go hand-in-hand, consistent with our prediction that dehumanization is related to harsher punishment. We also included a measure of liking of the offenders to ensure that the effects of dehumanization could not be accounted for by a general negative attitude toward them.

## Methods

### Participants and Design

A total of 120 individuals responded to a listing on the American Mechanical Turk website (63% women; 80% White, 6.5% Black, 9% Asian, 4.5% mixed or Native American; *M_age_* = 36.46, *SD* = 13.30, ranging from 18 to 76). This allowed for a reliable and diverse sample of respondents [53]. Inclusion criteria required that participants completed the survey. Participants were randomly assigned to read about either a violent crime (*n* = 46), white collar crime (*n* = 39), or a child molestation crime (*n* = 35).

### Materials and Procedure

The cover story and vignettes were identical to those used in Study 1, except we simplified the design by using only one description of each crime type. The moral outrage emotions and dehumanization questionnaires were identical to those used in Study 1. As noted above, we also included measures of blame and overall liking for each kind of offender. Study 2 also included additional questions assessing severity of recommended punishment for the offender.

#### Moral outrage

Participants completed questionnaires after reading their designated crime description. As in Study 1, the first questionnaire consisted of 10 emotion descriptors which comprised 3 different subscales measuring disgust (α = .92, *M* = 3.71, *SD* = 1.91), anger (α = .96, *M* = 4.53, *SD* = 1.84), and contempt (α = .94, *M* = 4.51, *SD* = 1.94) [47], [48].

#### Dehumanization

Participants were asked to consider the criminal in the description on the same 8-items used in Study 1.

Liking for offender: Three questions assessed global evaluations of the offender. This included “how much do you respect him”, “how much do you like him”, and “does he make a positive impression on you?” (1 = not at all; 7 = very much so; α = .91, *M* = 1.31, *SD* = 0.84).

#### Blame

Participants rated the extent to which the criminal should be “blamed for their actions” and how “morally responsible” the offender was (1 = not at all, 7 = extremely; α = .95, *M* = 6.36, *SD* = 1.14).

#### Punishment severity

Finally, participants indicated how many years sentence the criminal should receive (the maximum allowable entry was 99 years), and how harsh the sentence should be (1 = not at all harsh, 10 = very harsh). A final question assessed how suitable the criminal was deemed for a rehabilitation program (1 = not at all suitable, 10 = extremely suitable).

## Results

### Liking

A one-way ANOVA revealed there was a marginal effect of crime type on liking for the criminal, *F*(2,117) = 2.99, *p* = .054, η^2^ = .05 (violent crime: *M* = 1.54, *SD* = 1.12; white collar crime: *M* = 1.15, *SD* = 0.53; child molestation: *M* = 1.18, *SD* = 0.60). Participants liked the violent criminal more than the white collar criminal and the child molester. The measure of liking was related to sentence harshness (*r* = −.28, *p* = .002) and marginally related to length of jail sentence (*r* = −.16, *p* = .076), but it was unrelated to rehabilitation (*r* = .07, *p* = .426). These relationships remain when controlling for crime type (*r* = −.26, *r* = .14, *r* = .05). Controlling for liking did not alter the significant relationships between dehumanization and moral outrage or any of the punishment-related variables.

### Moral Outrage

**Figure 2:**
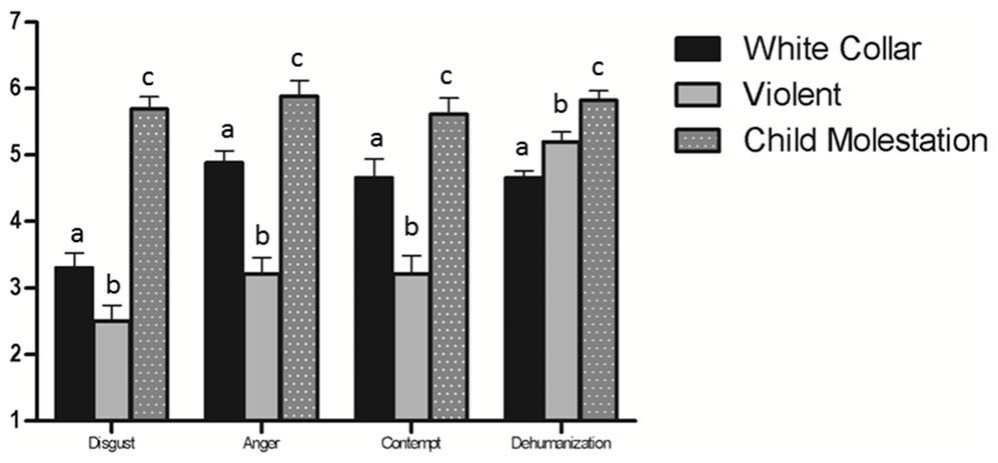
Mean differences in moral emotions and humanness across crime types, Study 2. Note: Values with different superscripts are significantly (*p*<.05) different from each other controlling for familywise error (Scheffé’s test).

A one-way ANOVA revealed a significant effect of crime type on each of the moral outrage emotions: Disgust, *F*(2,117) = 54.82, *p*<.001, η^2^ = .48; Anger, *F*(2,117) = 34.38, *p*<.001, η^2^ = .37; and Contempt, *F*(2,117) = 20.33, *p*<.001, η^2^ = .26. Post hoc comparisons (see Figure 2) again revealed that the child molestation crime produced more anger, contempt, and disgust compared to violent or white collar crimes. It is worth noting the differences in moral outrage to the white collar criminal in Study 2 compared to Study 1. In Study 2, the white collar criminal was the target of more disgust, anger, and contempt than the violent criminal than in Study 1. In Study 1 these differences, especially for anger and disgust, were in the other direction. A likely explanation for this variation between studies is that Study 2 was conducted in America in the aftermath of the global financial crisis, which was largely attributed to white collar criminal behavior. Study 1, on the other hand, was conducted in Australia which was relatively unaffected by the recent financial crisis and therefore moral outrage towards white collar crime was likely less salient.

### Dehumanization

Principal components analysis suggested a one factor solution explaining 41.31% of the variance. As with Study 1, we constructed a single dehumanization measure (α = .77, *M = *2.80, *SD = *1.12). A one-way ANOVA revealed a significant effect of crime type on dehumanization, *F*(2,117) = 11.84, *p<.*001, η^2^ = .17. Post hoc comparisons (see Figure 2) revealed that dehumanization was highest in the case of child molestation crimes, followed by violent crimes and white collar crimes.

### Correlates of Dehumanization

Table 1 shows the correlates of dehumanization for the entire sample. All of the moral outrage emotions were positively associated with dehumanization. However, in the case of disgust there was a disgust × crime type interaction, *b* = −0.98, *t*(116) = −2.40, *p* = .018. The relationship between disgust and dehumanization was significant for the child molestation crime, *r* = .71, *p*<.001, but not for the white collar (*r* = .12) or violent crimes (*r* = .00).

### Differences in Blame and Sentencing across the Three Types of Crimes

Table 2 shows mean differences in the punishment-related variables and blame. We log10-transformed responses to sentence length in years as this variable was negatively skewed (*M* = 14.79; *SD* = 20.71). All judgments significantly varied across conditions, with child molesters consistently viewed as deserving of harsher and longer jail sentences, and less suitable for rehabilitation. White collar criminals were seen as just as blameworthy and morally responsible as child molesters, but were sentenced less harshly and judged more suitable for rehabilitation.

**Table 2:** Means and standard deviations of variables as a function of crime type, Study 2.

	White Collar		Violent		Child Molestation		Significance Test
	(*n* = 40)		(*n* = 46)		(*n* = 36)		
	*M*	*SD*	*M*	*SD*	*M*	*SD*	
Blame	6.59^a^	1.02	5.90^b^	1.36	6.71^a^	0.70	F(2,115) = 6.78, p<.01, η^2^ = .10
Sentence harshness	7.28^a^	2.09	7.17^a^	2.07	8.94^b^	2.10	*F*(2,116) = 8.27, *p*<.001, η^2^ = .13
Jail sentence in years	7.77^a^	8.57	11.02^a^	16.27	27.57^b^	28.72	*F*(2,117) = 14.05, *p*<.001, η^2^ = .19
Rehabilitation	5.82^a^	2.75	5.52^a^	2.66	3.51^b^	3.01	*F*(2,117) = 7.40, *p*<.001, η^2^ = .11

NOTE: Within rows, values with different superscripts (a, b) are significantly (*p*<.05) different from each other controlling for familywise error (Scheffé’s test).

### Predictors of Punishment Severity

Table 3 shows the zero-order and partial correlations between blame, dehumanization, the moral outrage emotions, and the punishment-related variables, controlling for crime type (using dummy coded variables). Dehumanization was positively related to length of jail sentence, and sentence harshness. Conversely, it was negatively related to perceived suitability for rehabilitation. Importantly, dehumanization was also positively associated with perceptions of blame, rather than reduced blame. All moral outrage emotions were positively correlated with sentence length and were negatively related to suitability for rehabilitation; however, moral outrage emotions were unrelated to sentence harshness and blame when controlling for crime-type.

**Table 3:** Zero-order and partial correlations (controlling for crime type) between sentencing variables, blame, and dehumanization and moral emotions in Study 2.

	Blame	Jail sentence in years	Sentence harshness	Suitability for rehabilitation
	*r*	*r*	*r*	*r*
Blame		.11 (.19*)	.27** (.32***)	−.06 (−.13)
Dehumanization	.24** (.22*)	.28** (.40***)	.29*** (.37***)	−.24** (.34***)
Disgust	−.14 (.08)	.29*** (.48***)	.10 (.31***)	−.32*** (−.44***)
Anger	.06 (.24**)	.30*** (.41***)	.16 (.29***)	−.30*** (−.36***)
Contempt	.01 (.17)	.17† (.30***)	.08 (.21*)	−.32*** (−.39***)

NOTE: Zero-order correlations are in parentheses.

*** *p*<.001,

** *p*<.01,

* *p*<.05,

† *p* = .065.

Given the strong intercorrelations among the individual moral outrage emotions (Table 1), we collapsed across all three to form a measure of moral outrage (α = .95). Using a series of regression analyses, we then compared the effects of moral outrage and dehumanization in predicting punishment severity (see Table 4). This revealed that, controlling for crime type, moral outrage predicted sentence length and reduced suitability for rehabilitation independent of dehumanization; however, moral outrage did not predict sentence harshness. Dehumanization was a significant independent predictor of all sentencing variables, although only marginally so for rehabilitation suitability.

**Table 4:** Partial correlations and multiple regression analyses (controlling for crime type) predicting sentencing variables from dehumanization and moral outrage in Studies 2 and 3.

	Jail sentence in years	Sentence harshness	Suitability for rehabilitation
Study 2			
	*r*	*r*	*r*
Moral Outrage	.30***	.13	−.38***
	β	β	β
Moral Outrage	.30**	.09	−.43***
Dehumanization	.22*	.28**	−.18†
Study 3			
	*r*	*r*	*r*
Moral Outrage	.27***	.36***	−.30***
	β	β	β
Moral Outrage	.24**	.22**	−.22**
Dehumanization	.22**	.44***	−.33***

NOTE: ***p<.001,

** *p*<.01,

* *p*<.05.

† *p* = .054.

Finally, we note the positive correlation between blame and dehumanization (see Table 3) showing that in response to criminal behavior, dehumanization occurs in the context of increased (rather than reduced) blame. Noteworthy is the finding that dehumanization was more consistently related to punishment severity than blame, with blame only significantly related to sentence harshness. When both dehumanization and blame were entered into a simultaneous regression equation predicting sentence harshness, both remained significant predictors (Dehumanization: β = .32, *p*<.001; Blame: β = .26, *p* = .003). This indicates that although dehumanization is associated with increased blame, its relationship to punishment severity is not reducible to perceptions of blame.

## Discussion

Study 2 largely replicated Study 1, showing that moral outrage was associated with increased dehumanization irrespective of crime type (Hypothesis 1). Study 2 also demonstrated that moral outrage predicted two out of three measures of punishment severity (providing partial support for Hypothesis 2). In addition, dehumanization predicted punishment severity, and this occurred independently of any effects associated with moral outrage (Hypothesis 3).

Study 2 also extended the findings of Study 1 by showing the role of moral outrage and dehumanization in the severity of prescribed punishment. Dehumanization of offenders was consistently and positively related to harshness and length of sentencing. Moreover, dehumanization was negatively associated with perceived suitability for rehabilitation. This same pattern of relationships was evident for moral outrage, consistent with the findings of Carlsmith and Darley [21]; however, moral outrage did not predict sentence harshness. Critically, dehumanization also predicted severity of punishment, and did so irrespective of crime type and any effects associated with moral outrage, thereby providing an important extension of this previous work. Noteworthy is the additional finding that the relationships between dehumanization and moral outrage, and the punishment-related variables were not explained by differential liking of the offender. This suggests that our measure of dehumanization captured more than simple negativity. Finally, Study 2 also provided evidence that dehumanization was positively associated with perceptions of blame, although its relationship to punishment severity was not reducible to these perceptions.

Finally, it is noteworthy that although the individual moral outrage emotions were highly correlated and showed uniform relationships with dehumanization across crime-types, the dehumanization-disgust relationship was almost entirely driven by the child molestation vignette. This moderation by crime type is interesting in view of previous work suggesting strong links between dehumanization and disgust [42], [43], [54]. It suggests that disgust may be especially related to dehumanization in contexts where purity concerns are aroused (such as the protection of sexual innocence: see [55]).

### Study 3

Studies 1 and 2 provided support for all three of our hypotheses; however, there are still a number of factors not accounted for in these studies. First, we used different types of crimes in the first two studies, some clearly more serious than others. The focus on different crime types did not allow us to directly control for crime severity and crime seriousness. In Study 3, we focused solely on violent crimes but manipulated crime severity. We also obtained judgments of how much harm was caused to victims of the crime. This focus allowed us to determine the extent to which moral outrage and dehumanization remained significant predictors of punishment severity when there were no qualitative differences in the types of crimes. Second, we also tested the possibilities that moral outrage and dehumanization would mediate the effects of judgments of the harmfulness on the severity of the punishment (Hypotheses 4 and 5, respectively).

## Methods

### Participants and Design

A total of 166 individuals responded to a listing on the American Mechanical Turk website. As in Study 2, this recruitment method allowed for a reliable and diverse sample of respondents [53]. Inclusion criteria required that participants completed the survey. In order to ensure compliance with instructions, at the end of the study, within the Ten-Item Personality Inventory [56], we imbedded a manipulation check asking participants to give a specific response (i.e., please click “agree a little”). Ten participants failed this check, which left 156 participants (48%% women; 77% White, 9% Black, 7% Asian, 3% Hispanic, 4% other; *M_age_* = 33.32, *SD = *11.15, ranging from 18 to 70). Participants were randomly assigned to read about 1 of 4 violent crimes which increased in seriousness, from threatening others (*n = *39), to assaulting others (*n = *37), to attacking and seriously injuring others (*n = *41), to killing others (*n* = 39).

### Materials and Procedure

The vignettes were based on examples taken from the The National Survey of Crime Severity [57]. This allowed us to carefully control for crime severity. The vignettes described a violent crime (see Appendix S2). Participants completed questionnaires after reading their designated crime description.

#### Moral outrage

As in Study 1 and Study 2, the first questionnaire consisted of 10 emotion descriptors which comprised 3 different subscales measuring disgust (α = .87, *M = *4.12, *SD = *1.73), anger (α = .97, *M = *4.69, *SD = *1.97), and contempt (α = .90, *M = *4.55, *SD = *1.86) [47], [48].

#### Dehumanization

Participants were asked to consider the criminal in the description on the same 8-items used in Studies 1 and 2.

#### Liking for offender

The same three questions used in Study 2 assessed global evaluations of the offender. This included “how much do you respect him”, “how much do you like him”, and “does he make a positive impression on you?” (1 = not at all; 7 = very much so; α = .91, *M* = 1.27, *SD* = 0.78).

#### Crime harmfulness

Participants were asked to rate how much harm had been caused to the victims of the crime (1 = none, 7 = a lot; *M* = 6.13, *SD* = 1.41).

#### Sentencing

Finally, as in Study 2 participants indicated how many years of sentencing the criminal should receive, and how harsh this sentence should be (1 = not at all harsh, 10 = very harsh; *M* = 8.91, *SD* = 1.76), and how suitable the criminal was deemed for a rehabilitation program (1 = not at all suitable, 10 = extremely suitable; *M* = 3.39, *SD* = 2.76).

## Results

### Liking

A one-way ANOVA revealed no effect of crime severity on liking for the criminal, *F*(3,152) = 1.15, *p* = .330, η^2^ = .02 (threatening others: *M* = 1.34, *SD* = 0.89; assaulting others: *M* = 1.29, *SD* = 0.84; attacking and seriously injuring others: *M* = 1.37, *SD* = 0.93; killing others: *M* = 1.08, *SD* = 0.26). The measure of liking was significantly related to all three punishment variables (sentence harshness, *r* = .61, *p*<001; sentence length, *r* = .42, *p*<.001; rehabilitation, *r* = −.33, *p*<.001) and this remained when controlling for crime severity (*r* = .62, *r* = .45, *r* = −.32, respectively). Controlling for liking did not alter the significant relationship between dehumanization any of the moral outrage emotions, and did not alter the significant relationship between dehumanization and any of the punishment related variables.

### Moral Outrage

A one-way ANOVA revealed a significant effect of crime severity on Disgust, *F*(3,152) = 2.93, *p* = .035, η^2^ = .06; however, post hoc analyses showed no significant differences between conditions. There was no effect of crime severity on Anger, *F*(3,152) = 2.03, *p* = .112, η^2^ = .04 or Contempt, *F*(3,152) = .63, *p* = .599, η^2^ = .01.

### Dehumanization

Principal components analysis indicated a one factor solution explaining 35.94% of the variance. As with Studies 1 and 2, a single dehumanization measure was constructed (α = .70, *M = *2.31, *SD = *0.89). A one-way ANOVA revealed that there was no effect of crime severity condition on dehumanization, *F*(3,152) = .12, *p* = .949, η^2^ = .01.

### Correlates of Dehumanization

Table 1 shows the correlates of dehumanization and the moral outrage emotions for the entire sample. All of the moral outrage emotions were positively associated with dehumanization.

### Differences in Punishment Severity

We log10-transformed responses to sentence length in years as this variable was negatively skewed, with participants recommending between 0 and 99 years as jail terms (*M* = 55.63, *SD* = 40.71). One-way ANOVAs revealed a significant effect of crime severity on length of jail sentence, *F*(3,152) = 43.19, *p*<.001, η^2^ = .46, sentence harshness, *F*(3,152) = 10.14, *p*<.001, η^2^ = .17, and whether the perpetrator was suitable for rehabilitation, *F*(3,152) = 14.74, *p*<.001, η^2^ = .23. The same ANOVA also revealed an effect of crime severity on judgments of the harmfulness of the crime, *F*(3,152) = 32.99, *p*<.001, η^2^ = .39 (see Table 5 for post hoc comparisons).

**Table 5:** Means and standard deviations of variables as a function of crime severity in Study 3.

	Threatened		Assaulted		Injured		Killed		Significance Test
	(*n* = 39)		(*n* = 37)		(*n* = 41)		(*n* = 39)		
	*M*	*SD*	*M*	*SD*	*M*	*SD*	*M*	*SD*	
Harmfulness	4.64^a^	1.78	6.41^b^	1.01	6.58^b^	0.77	6.90^b^	0.78	*F*(3,152) = 32.99, *p*<.001, η^2^ = .39
Sentence harshness	7.72^a^	2.08	9.24^b^	1.72	9.10^b^	1.51	9.61^b^	1.02	*F*(3,152) = 10.14, *p*<.001, η^2^ = .17
Jail sentence in years	15.38^a^	2.87	64.41^b^	35.34	59.44^b^	37.71	83.56^b^	27.91	*F*(3,152) = 43.19, *p*<.001, η^2^ = .46
Rehabilitation	5.54^a^	2.87	2.41^b^	1.95	3.32^b^	2.69	2.25^b^	2.15	*F*(3,152) = 14.74, *p*<.001, η^2^ = .23

NOTE: Within rows, values with different superscripts (a, b) are significantly (*p*<.05) different from each other controlling for familywise error (Scheffé’s test).

### Predictors of Punishment Severity

Table 6 shows the partial correlations between the punishment-related variables, dehumanization, and the moral outrage emotions controlling for crime severity. Harmfulness, dehumanization, and all moral outrage emotions were positively related to length of jail sentence, and sentence harshness, and negatively related to perceived suitability for rehabilitation.

**Table 6:** Zero-order and partial correlations (controlling for crime severity in parentheses) between sentencing variables, crime judgments, dehumanization and moral emotions in Study 3.

	Harmfulness	Jail sentence in years	Sentence harshness	Suitability for rehabilitation
	*r*	*r*	*r*	*r*
Harmfulness		.67*** (.77***)	.69*** (.73***)	−.61*** (−.67***)
Dehumanization	.42*** (.37**)	.35*** (.31**)	.55*** (.53***)	−.42*** (−.41***)
Disgust	.26*** (.32***)	.19** (.26***)	.27*** (.31***)	−.21** (−.26***)
Anger	.22** (.29***)	.21** (.28***)	.30*** (.34***)	−.20* (−.26***)
Contempt	.27** (.28***)	.29*** (.30***)	.35*** (.36***)	−.35*** (−.37***)

NOTE: Zero-order correlations are in parentheses.

*** *p*<.001,

** *p*<.01,

* *p*<.05.

Given the strong intercorrelations among the individual moral outrage emotions, as in Study 2 we collapsed across all three to form a measure of moral outrage (α = .93). When moral outrage and dehumanization were entered into multiple regression models, both variables independently predicted all three measures of punishment severity.

We next used Preacher and Hayes’ [58] syntax to determine whether moral outrage and dehumanization mediated the relationship between perceived crime harmfulness and punishment severity (see Table 7). In Model 1, we tested the mediating effects of moral outrage. This revealed that moral outrage was a significant mediator of the relationship between harmfulness and sentence harshness, and also the relationship between harmfulness and suitability for rehabilitation. In Model 2, we tested the mediating effects of dehumanization on this same relationship. This revealed that dehumanization was also a significant mediator of the relationship between harmfulness and sentence harshness and suitability for rehabilitation. In Model 3, we entered moral outrage and dehumanization as mediators. This revealed the dehumanization, but not moral outrage, remained a significant mediator in the model. There were no mediation effects for sentence length.

**Table 7:** Bootstrapping test of mediation effects of moral outrage and dehumanization on the relationship between crime harmfulness and severity of punishment using Preacher and Hayes (2008) syntax in Study 3.

	Jail sentence in years	Sentence harshness	Suitability for rehabilitation
	*t*	*t*	*t*
All Models			
Harmfulness → Punishment (c)	13.76***	13.39***	−11.27***
Harmfulness → Moral outrage	4.56***	4.56***	4.56***
Harmfulness → Dehumanization	4.98***	4.98***	4.98***
Model 1– Moral outrage			
Moral outrage → Punishment	1.24	2.86**	−2.02
Harmfulness → Punishment (c′)	13.76***	11.87***	−9.98***
Bootstrap Results	−.01,.10	.03,.15	−.21, −.01
Model 2– Dehumanization			
Dehumanization → Punishment	.50	5.47***	−2.98**
Harmfulness → Punishment (c′)	13.78***	11.51***	−9.61***
Bootstrap Results	-.03,.07	.07,.23	−.26, −.03
Model 3– Moral outrage & Dehumanization			
Moral outrage → Punishment	1.13	1.42	−1.19
Dehumanization → Punishment	.13	4.78***	−2.47*
Harmfulness → Punishment (c′)	13.16***	10.91***	−9.08***
Bootstrap Results (Moral Outrage)	−.02,.06	−.01,.24	−.17,.04
Bootstrap Results (Dehumanization)	−.05,.06	.05,.24	−.24, −.01

NOTE: ***p<.001,

** *p*<.01,

* *p*<.05.

## Discussion

Study 3 replicated the findings of Studies 1 and 2 by showing that moral outrage and dehumanization co-occurred in response to criminal behavior (Hypothesis 1). Study 3 also provided further evidence that moral outrage and dehumanization were associated with the severity of recommended punishment (Hypotheses 2 and 3). Finally, Study 3 also provided support for the mediating effects of moral outrage (Hypothesis 4) and dehumanization (Hypothesis 5) on the relationship between the perceived harmfulness of the crime and the severity of the recommended punishment. Moreover, consistent with our prediction, the mediating effects of dehumanization were independent of any effects associated with moral outrage (Hypothesis 5). We only observed this mediation for the harshness of the sentence and perceived lack of suitability for rehabilitation. The relationship between the harmfulness of the crime and the length of the sentence was not mediated by either moral outrage or dehumanization. It is possible that this may reflect the more rational, or non-emotive, nature of this recommendation. In contrast, claiming that the sentence should be harsh and that the offender is unlikely to be rehabilitated may be driven by more emotional and reactive factors. If so, this notion may explain why moral outrage and dehumanization played a mediating role in the relationship between these judgments and the perceived harmfulness of the crime.

## General Discussion

Across three studies we found support for the influences of moral outrage and dehumanization on severity of offender punishment. As predicted, moral outrage and dehumanization also covaried across crime types (Studies 1 and 2) and crime severity (Study 3) and predicted sentencing judgments independent of crime type and crime severity (Studies 2 and 3). Moral outrage and dehumanization also partially explained how the perceived harmfulness of a crime is translated into recommendations for harsh or severe punishment, and the perception that the offender is not amendable to rehabilitation.

Our findings both support and extend Carlsmith et al.’s [23] retributive justice model. Consistent with their approach to understanding retributive justice, we found an association between moral outrage and endorsement of more severe punishment. Moreover, we also showed that morally outraged individuals viewed the offender as unlikely to be rehabilitated. Most importantly, however, we extended this work by highlighting an important role for dehumanization of the offender. People are not only morally outraged by harmful and violent crimes against others, but also view the perpetrators of these crimes as subhuman and beastly. Consistent with the work of Viki et al. [33], this perception was associated with harsher and longer punishment and a perception that the offender is unsuitable for rehabilitation. Critically, however, we also found that dehumanization partially accounted for how perceptions of crime harmfulness were translated into a desire for severe forms of punishment. This novel finding supports the addition of dehumanization to models of retributive justice. We even found this effect of dehumanization to be evident when accounting for moral outrage.

Our findings also identify for the first time a link between moral outrage and dehumanization. These two responses to criminal behavior co-occurred independently of crime type or crime severity. Although the causal direction of this relationship cannot be determined from the current data, one might expect that moral emotional responses are primary [24], with perceptions of the qualities of the perpetrators secondary to these responses. However, other causal relations between these variables are possible and future research could examine bidirectional effects.

It is important to note that our approach to measuring moral outrage in some ways differs from that used in previous work. We conceptualized moral outrage as a collection of emotional reactions to criminal behavior (cf., [26]) whereas past work has sought to differentiate the effects of these different emotional responses on social judgments. Disgust has been linked to norm violations related to sexuality, the body, and to concerns over purity [27], [48], [59]. Some work suggests that these disgust responses are relatively independent of responses involving anger triggered by perceptions of intentional harm [60], [61]. Contempt has also been conceptualized as separate from anger and disgust [27], [62]. Although we acknowledge the different functions of each of these emotions, they correlated highly in our studies (mean *r* = .62), and our main focus was to observe generalized moral emotional reactivity to criminal behavior. Future research may manipulate individual moral emotions in order to better differentiate between these emotional responses.

Finally, the results of our studies add to a body of work showing that dehumanization may be associated with harsher punishment [9], [28] and that this effect of dehumanization extends to criminal behavior [33]. This is in contrast to other work showing that certain forms of dehumanization may be associated with less blame [14], [16], [29], a possibility that sits comfortably with the fact that under many legal codes the mentally ill, a frequently dehumanized group, are given more lenient sentences than healthy individuals.

We believe that this tension can be resolved by understanding two potential ways that people can be dehumanized. When dehumanization arises in response to criminal behavior, it is likely to be associated with the individual’s moral character (e.g., “only an animal would do a thing like that”), leaving their responsibility undiminished. This interpretation is supported by our finding that dehumanization was negatively related to perceived suitability for rehabilitation: people who intentionally commit inhuman acts are not considered good candidates for intervention. Conversely, when dehumanization is related to antecedent conditions considered to be responsible for criminal behavior (e.g., underlying mental illness) it is likely to diminish perceived responsibility for the behavior, but leave the individual’s moral character intact. Notably, in such cases rehabilitation (i.e., mental health treatment) is often the preferred option.

In sum, our findings highlight that offender dehumanization is associated with moral outrage and may play an important role in determining the severity of punishment. Our findings provide important insights into processes of retributive justice [20]. When intentional harm is done others, people are motivated by “just deserts” concerns, seeking to punish offenders in accordance with what they deserve. In addition to reacting to the harm done with moral outrage, people also view the offender as lacking core human qualities. In the eyes of third party observers, this perception of criminals as subhuman and beastly therefore makes them more deserving of severe and coercive forms of punishment and as less capable of rejoining society.

## Supporting Information

Appendix S1 **Crime vignettes used in Studies 1 and 2.**
(DOCX)

Appendix S2 **Crime vignettes used in Study 3.**
(DOCX)
