# Supplementary material for: The Roles of Dehumanization and Moral Outrage in Retributive Justice
Source: PLoS One. 2013 Apr 23;8(4):e61842. doi: 10.1371/journal.pone.0061842 (PMC3633929; doi:10.1371/journal.pone.0061842)
Supplement: Appendix S1 — Crime vignettes used in Studies 1 and 2. (DOCX) [file pone.0061842.s001.docx]

**Appendix S1**

*Violent crime 1:* Ryan Macey is a 41 year old Caucasian man from Sydney. On a Tuesday afternoon, he attempted to hijack a bus carrying 50 passengers by threatening the driver with a knife. Macey boarded the bus stopped in traffic, forced the driver off, and told passengers to stay put. He failed to start the bus before off-duty Senior Constable John Rider arrived. Macey lunged at the officer with a chisel before fleeing towards the Westfield shopping centre, where police lost track of him. Ryan was shirtless and seen on TV soon after the crime. He was arrested about 2.35pm when a resident phoned police saying they had been threatened by a man hiding out in their garage, and was found carrying a bum-bag containing a chisel, screwdriver, scissors and a 20 centimetre blade knife.*

*Violent crime 2:* Xinhua Wu is a 48 year old landlord who hacked to death seven young children and two adults with a meat cleaver at a kindergarten in rural Victoria. Wu had rented a house to the proprietor of the privately run kindergarten and was upset that the property was not vacated in April, when the school's lease expired. Xinhau Wu carried out the attacks at the start of a school day before being taken into custody.

*White collar Crime 1:* James Scott is a 34 year old, Caucasian man who lives in Greenslopes in Brisbane’s south. He fleeced almost $127,000 from family and friends by stealing money they had given to him for investments. He stole this large amount of money from 6 clients between February 2002 and July 2004. He had encouraged clients, family and friends to invest in shares, managed investments and superannuation funds. Instead of investing the money, Scott transferred the funds into his own accounts and used the money for personal expenses. Scott has been sentenced to 10 month s in jail before being released on a five year good behaviour bond. *

*White collar crime 2:* David Menzies, a 27 year old Australian man was arrested in Las Vegas and charged for laundering $540 million in online gambling proceeds. Between February 2008 and March 2009, Menzies’ company allegedly processed more than $540 million in transactions between US gamblers and internet gambling websites, disguising transactions to the banks so they would appear unrelated to gambling. Menzies then arranged for the funds to be wired offshore for the benefit of the gambling companies. Daniel now faces a maximum sentence of 75 years in prison for bank fraud, money laundering, gambling conspiracy and processing electronic funds illegally.

*Child Molester 1:* Mathew Bronson is a 44 year old Caucasian man from Brisbane. He has recently sexually molested a 4 year old girl in her house at Kangaroo Point, in inner Brisbane. Bronson broke into the four year olds house on March 26, had removed her pants and sexually abused her before he was interrupted by a family member entering the room. Bronson jumped out the window and fled the scene, but was found by one of the girl’s relatives a short time later. He was sentenced to 4 years’ jail.*

*Child Molester 2:* Simon Schwartz, a 53year old paediatrician from Perth used his practice to molest boys and teenagers for more than 20 years. Stories about the incidents emerged following a two-year investigation into allegations that Dr Schwartz was providing improper prescriptions to minors. Swartz performed oral sex on at least two young men when they were teenagers and paid them $200, while he fondled a third nine year-old boy’s genitals. A home video shot by Dr Schwartz was also found featuring adult discussions with teenage boys. Dr Schwartz is awaiting trial without bail.

*Denotes vignettes used in Study 2.
