# Supplementary material for: The Roles of Dehumanization and Moral Outrage in Retributive Justice
Source: PLoS One. 2013 Apr 23;8(4):e61842. doi: 10.1371/journal.pone.0061842 (PMC3633929; doi:10.1371/journal.pone.0061842)
Supplement: Appendix S2 — Crime vignettes used in Study 3. (DOCX) [file pone.0061842.s002.docx]

**Appendix S2**

*Violent crime 1:* Jacob White is a 40 year old landlord who **threatened** seven young children and two adults with a meat cleaver at a kindergarten in rural Victoria. White had rented a house to one of the adult victims.

*Violent crime 2:* Jacob White is a 40 year old landlord who **assaulted** seven young children and two adults with a meat cleaver at a kindergarten in rural Victoria. White had rented a house to one of the adult victims.

*Violent crime 3:* Jacob White is a 40 year old landlord who **attacked and seriously injured** seven young children and two adults with a meat cleaver at a kindergarten in rural Victoria. White had rented a house to one of the adult victims.

*Violent crime 4:* Jacob White is a 40 year old landlord who **hacked to death** seven young children and two adults with a meat cleaver at a kindergarten in rural Victoria.  White had rented a house to one of the adult victims.

Note: Bold font did not appear in the actual study materials.
